# Risk of Developing Hepatocellular Carcinoma following Depressive Disorder Based on the Expression Level of Oatp2a1 and Oatp2b1

**DOI:** 10.1155/2019/3617129

**Published:** 2019-07-29

**Authors:** Yan Chen, Jiongshan Zhang, Mengting Liu, Zengcheng Zou, Fenglin Wang, Hao Hu, Baoguo Sun, Shijun Zhang

**Affiliations:** ^1^Department of Traditional Chinese Medicine, The First Affiliated Hospital, Sun Yat-sen University, 58, Zhongshan Road II, Guangzhou, 510080, China; ^2^Department of Traditional Chinese Medicine, The Third Affiliated Hospital, Sun Yat-sen University, Guangdong Key Laboratory of Liver Disease Research, 600, Tianhe Road, Guangzhou, 510630, China

## Abstract

**Background:**

Accumulating evidence from prospective epidemiological studies has showed that depression disorder (DD) is a risk factor for cancer. The aim of this study is to explore the association of DD and the overall occurrence risk of hepatocellular carcinoma (HCC) and the mechanism.

**Methods:**

In this study, 60 mice were randomly divided into four groups: Control group, DD group, HCC group, HCC-DD group. Mice received a chronic dose of reserpine to establish depression model, followed by Diethylnitrosamine and Carbon tetrachloride administration to establish HCC models. Behavioral depression was assessed by sucrose preference test (SPT) and the expression of Serotonin 1A (5-HT1A) receptor in the hippocampal. The expression of Oatp2a1 and Oatp2b1 in the digestive system tissues was detected by PCR and western blotting.

**Results:**

Reserpine-administrated mice had a reducing sucrose preference at Day 14 compared with blank mice (P<0.05). The expression of 5-HT1A receptor in the hippocampal was decreased in DD mice compared with blank mice. The survival analysis indicated that the HCC mice with DD have poorer survival rate compared with the HCC mice. Compared with HCC mice, the expression of Oatp2a1 and Oatp2b1 was lower in liver and stomach tissue and higher in hepatic carcinoma and colon tissue of HCC-DD mice (P<0.05), and the expression of Oatp2a1 was higher in the spleen tissue of HCC-DD mice while the expression of Oatp2b1 was lower (P<0.05). However, no difference was found in the expression of Oatp2a1 and Oatp2b1 in the small intestine tissue between HCC group and HCC-DD group.

**Conclusions:**

DD was the adverse factors for the overall occurrence risk of HCC. Mechanistically, be the downregulation of Oatp2a1 and Oatp2b1 in liver tissue induced by DD might be involved.

## 1. Introduction

Globally, HCC is the fifth most common cancer and is the second cause of cancer mortality [1]. Statistics shows a rising incidence of HCC from 1.5 per 100,000 persons to nearly 14 per 100,000 persons, and a 5‐year survival under 12% in the last 40 years even in the low incidence of hepatitis B in the United States [2]. Although liver resection, transplantation, radiofrequency ablation (RFA), or even for ablative techniques of transcatheter arterial chemoembolization (TACE) have been improved and applied widely for HCC, HCC recurrence remains to be a challenge [3]. The molecular targeted therapies are the only treatment modalities for patients with advanced HCC. Sorafenib provides a meaningful survival benefit in patients with advanced HCC; however, as other molecular targeted drugs developed, they still have serious side effects [4]. Therefore, increasing researchers focus on exploring the pathogenic factors and etiopathogenesis of HCC, aiming at provide suitable treatment or even preventing HCC.

Studies suggested that the long time chronic social defeat stress induced by life style changing contributes to the pathogenesis of major DD [5]. An interesting study demonstrated that the increasing comparisons on Facebook may induce feelings of envy, which may be related to DD [6]. DD is not only the leading cause of decrement in health utility in general population, but also in the cancer patients [7]. The researchers have found that hepatoma-burden might induce depressive-related behavior and antidepressants might not only palliate depression symptoms but also modify disease processes in the auxiliary treatment of cancer. Moreover, the antidepressants of selective serotonin reuptake inhibitors (SSRIs) may possibly reduce the risk of HCC in HBV-infected patients in a dose-responsive manner [8, 9].

Recently, growing evidence from prospective epidemiological studies has showed that DD is a risk factor for cancer. The study suggested that DD may be related to increased risk of ovarian cancer [10]. Gut-derived serotonin induced by depression could promote breast cancer bone metastasis through the RUNX2/PTHrP/RANKL pathway in mice, and DD might be responsible for progression of malignancy and thus accelerate the metastasis formation in cancer patients [11]. Another study showed a close relationship between DD and the overall occurrence risk of HCC without mentioning its underlying mechanism [12]. And the relationship between DD and HCC has been seldom reported so far.

Organic anion transporters (Oatps) as a superfamily of transporter proteins is globally expressed throughout tissues and organs of human body with different abundance and the organs and tissues of digestive system no exception [13]. Oatp family proteins mainly carry out the uptake of small molecules and exert important functions to maintain the homeostasis of human body under physiological condition [14]. Oatp proteins in digestive system are involved in absorbing nutrients and ions, excreting bile acids, and metabolise toxins [14–18]. Thus, dysfunction of Oatp proteins in digestive system may easily mediate the initiation and progression of tumors [19]. The study reported that Oatp2a1 is likely to promote tumorogenesis by PGE2 uptake into the endothelial cells; blockade of Oatp2a1 is an additional pharmacologic strategy to improve colon cancer outcomes [20]. The expression of Oatp2b1 mRNA was increased in bone tumors [21]. Oatp2 is mainly expressed on the sinusoidal membrane of human hepatocytes and transporting both unconjugated and conjugated bilirubin from plasma into the liver [22]. In liver, Oatp2 is in charge of the uptake and glucoronidation of bilirubin in hepatocytes; mutation of Oatp2 may be involved in the development of hyperbilirubinemia [23]. It plays an important role in the excretion of bilirubin and liver toxin [24]. We hypothesize that, as the important members of transporter proteins to excrete bilirubin and liver toxin, Oatp2a1 and Oatp 2b1 may also be relevant to HCC.

Recently, the disordered tissue microenvironment and imbalanced internal environment which is further enhanced by the causes of metabolic have been proposed as the pathogenic factors of HCC [25]. DD, caused by the stress from physiological, psychological, and physical factors, has an adverse impact on human body homeostasis [26]. To our knowledge, DD has an adverse impact on human body homeostasis and has a relationship with HCC, Oatp 2a1, and Oatp 2b1 as the important members of transporter proteins in the internal environment are closely related with the occurrence of digestive system tumors; internal environment disorder is the important factor for HCC. Therefore, we will observe the expression of Oatp 2a1 and Oatp 2b1 on HCC-DD mice and explore the relationship between DD and HCC at the molecular level in body internal environment in this study.

## 2. Materials and Methods

### 2.1. Experimental Animals

In total 60 specific pathogen-free (SPF) C57BL/6 mice (male; weight, 18±2g; series No.: 44008500008530) were purchased from the Animal Experimental Center of Sun Yat-sen University (Certification No.: SYXK (Guangdong) 2012-0081; Guangzhou, China).

### 2.2. Chemicals

Reserpine injection (lot number: 1210111) was purchased from Jinyao Amino Acids Co., Ltd., (Tianjin, China). Diethylnitrosamine (CAS 55-18-5, N-nitrosodiethylamine, DEN) was purchased from Sigma-Aldrich Shanghai Trading Co., Ltd., (Shanghai, China). Carbon tetrachloride (CAS 56-23-5, CCl_4_) and olive oil (CAS 8001-25-0) were purchased from Aladdin biological technology co., Ltd., (Shanghai, China). Rabbit polyclonal to 5HT1A Receptor antibody (ab85615) was provide by Abcam Co., Ltd., (USA). Goat anti-rabbit IgG-HRP (sc-2004) was provided by Santa Cruz Biotechnology, Inc. (USA).

### 2.3. Experimental Design

After one-week acclimatization, sixty mice were randomly divided into four groups as follows (n=15 per group): (A) Control group, (B) DD group, (C) HCC group, and (D) HCC-DD group. At phase one, thirty mice (Group B and Group D) were accepted DD model procedure; then sixty mice (the all groups) were evaluated by Sucrose Preference Test at the same time. At the second phase, fifteen blank mice (Group C) and fifteen DD animals (Group D) were used to establish HCC model one week after phase one.

### 2.4. Induction of DD Animal Model

A chronic dose of reserpine administration was used to establish the animal model of DD according to the previous study [27]. The mice received a daily dose of reserpine (0.28 mg/kg i.p.) for 14 days; then the DD models were evaluated by Sucrose Preference Test.

### 2.5. SPT

Three days before the reserpine administration, the animals were placed in a cage with two bottles of 1% sucrose solution for 24 h to serve as a baseline. Then animals were presented with a 1% sucrose solution bottle and a water bottle for 24 h and two water bottles for another 24 h in order to adapt. Sucrose preference test was carried out on the 7th day and the 14th day. Animals were free to access two bottles containing 1% sucrose solution and water for 24 h, respectively. The consumed sucrose solution and water was recorded. The sum of water consumption and sucrose consumption was defined as total intake. The percentage of sucrose intake was calculated using the following equation: % sucrose preference= sucrose intake×100/total intake [28].

### 2.6. Induction of HCC in Animal Model

HCC was induced by four steps. Firstly, animals received single (i.p.) injection of DEN (95 mg/kg) diluted in saline (100 mg/ml). After that, promotion was induced by intragastric administration of CCl_4_ as a single necrogenic dose (5 ml/kg diluted in olive oil at a ratio of 1:4) twice a week from the fourth day. Then all the animals were intraperitoneal injection of diluted DEN once with a reduced dose 50 mg/kg at the third week. Finally, animals had intragastrical administration by the increased dose of CCl_4_ (8 ml/kg, diluted in olive oil at a ratio of 1:4) twice a week from the fourth week until the 20 weeks. This was followed by feeding the animals the basal diet till the end of the study [29].

### 2.7. Sample Collection

Mice were sacrificed by cervical dislocation after they were anesthetized using 10% chloral hydrate (3.5 ml/kg, i.p.); all the brains, the livers, the spleens, the stomachs, the small intestines, and the colons were collected. One part of the samples were stored at −80°C for the following researches; the other parts of the samples were fixed with 4% paraformaldehyde and then embedded in paraffin for immunohistochemistry. Meanwhile, when the mice reached the ethical limits of animal care (the body weight loss 20%, chills or ascites), they were sacrificed by cervical dislocation under anesthesia immediately in order to relieve suffering and the samples were collected.

### 2.8. Immunohistochemistry (IHC)

The hippocampi were separated from the brains which were fixed with 4% paraformaldehyde. Then, the hippocampi tissues were dehydrated and set in paraffin. After dewaxing by xylene, ethanol, and distilled water, the sections were put into citrate buffer solution and heated in a microwave oven. The slides were blocked using 6.5% BSA at room temperature and incubated in diluted primary antibody (1:500) at 4°C overnight. After washing thrice in PBS, the sections were incubated with diluted secondary antibody (1:1,000) for 1 h at room temperature. After washing with distilled water the sections were counterstained with Harris hematoxylin solution dehydrated, cleared in xylene, and mounted with synthetic resin mounting medium and coverslip. Brown-yellow stain was positive staining [30].

### 2.9. Fluorescence-Based Quantitative RT-PCR Reactions

The levels of Oatp 2a1 and Oatp 2b1 mRNA in the digestive system tissues were analyzed by Real-Time quantitative-PCR. The total RNA was extracted using Trizol, and the DNA was obtained by reverse transcription with a BIO-RAD quantitative PCR instrument. The quantitative standard curve was prepared based on a gradient of positive standard with a negative control of sterile double-distilled water. The following probes and primers were used for mRNA analysis: Oatp2a1:forward primer:5′-CTC CCG TCC ATC TTC CTC ATC T-3′, reverse primer:5′-AGA ACT GTA CTC CAA TGG CAA ACG A-3′; Oatp2b1:forward primer:5′-GGT GGC TGG GCT TCC TCA TCT-3′, reverse primer: 5′-CCA AGA CCT TCC GCC TGA AAT GA-3′; GAPDH:forward primer:5′-AGA AGG TGG TGA AGC AGG CAT C-3′, reverse primer:5′-CGA AGG TGG AAG AGT GGG AGT TG-3′. The PCR conditions were as follows: 94°C for 30s was operated for 1 cycle, then 94°C for 5 s, 55°C for 15 s, and 72°C for 10 s were repeated for 40 cycles, and after that 60°C for 15 s and 95°C for 15 s were operated for 1 cycle.

### 2.10. Western-Blotting

The Oatp 2a1 and Oatp 2b1 protein expression in the digestive system tissues were analyzed by western blot. Tissue lysates were clarified by centrifugation at 4°C, 12000r/min for 30 minutes. Protein extract was separated on 10% sodium dodecyl sulphate-polyacrylamide gel electrophoresis. And then it was electrophoretically transferred onto a PDVF membrane (Millipore, Etten-Leur, The Netherlands) for 90 min at 100V. Subsequently, the PVDF membrane which had been blocked in 5% nonfat milk for 1 h at room temperature was incubated with primary antibodies. The primary antibodies Slco2a1(Santa Cruz biotechnology, USA) and Slco2b1(Abcam, USA), GAPDH (ABclonal Biotechnology, USA), were diluted in 1:1000, 1:500 and 1:5000, respectively at 4°C overnight;. And the secondary antibody Horseradish peroxidase (HRP) (Zhongshanjinqiao Biotechnology, Beijing, China) was diluted in 1:5000 at room temperature for 1 h. The membranes were washed with TBST, and the proteins were analysed by using ECL chemiluminescence.

### 2.11. Statistical Analysis

The data were analyzed using the Statistical Product and Service Solutions (SPSS17.0) software package for Windows. The survival time was analyzed using the Kaplan-Meier and Log-Rank (Mantel-Cox) test. The normally distributed data were analyzed using an independent t-test. A Wilcoxon nonparametric test was used while normal distribution wasnot assumed. P<0.05 was considered to indicate a statistically significant difference.

## 3. Results

### 3.1. Effects of the DD Model Established by Reserpine

**Figure 1:**
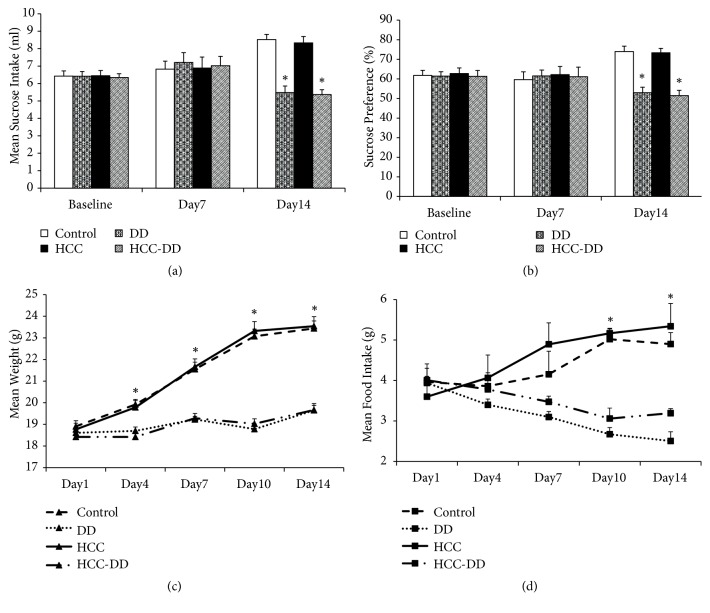
Sucrose preference test. Control, Control group; DD, DD group; HCC, HCC group; HCC-DD, HCC-DD group. ^*∗*^*P*<0.05 vs. Control group and HCC group. (a) Sucrose intake; (b) sucrose preference; (c) body weight; (d) food intake.

Sucrose preference test is an important method to assess behavioral depression. Reserpine-administrated mice (DD group and HCC-DD group) did not significantly differ from blank mice (Control group and HCC group) in sucrose preference at Day 7 but had a reducing sucrose preference at Day 14 compared with blank mice (P<0.05, Figures 1(a) and 1(b)). Between-group analyses showed that reserpine-exposed animals weighed significantly less compared to blank animals through Day 4 till Day 14 (P<0.05, Figure 1(c)). In addition, the data of food intake of DD animals was also less compared to blank animals through Day 4 till Day 14 (P<0.05, Figure 1(d)).

**Figure 2:**
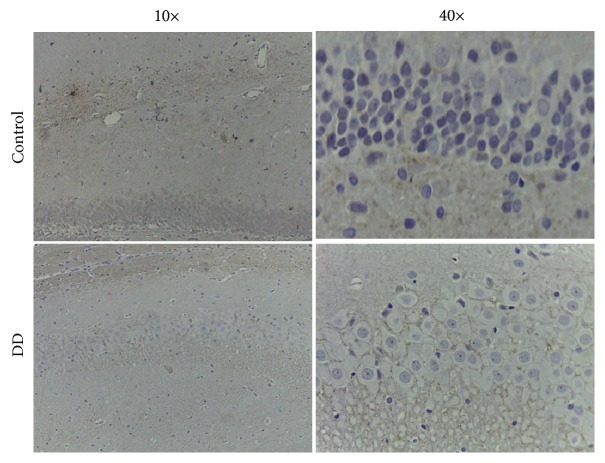
Change in the hippocampal 5-HT1A receptor expression of mice by immunohistochemistry (IHC).

5-HT1A receptor of the hippocampal is an admittedly important biological marker of DD. The expression level of 5-HT1A receptor in the CA1 region of the hippocampus was detected by IHC. DD triggered the decrease in 5-HT1A receptor expression in the CA1 region in the hippocampus compared with blank mice without DD (Figure 2).

### 3.2. Pathological Analysis of Hepatic Carcinoma

**Figure 3:**
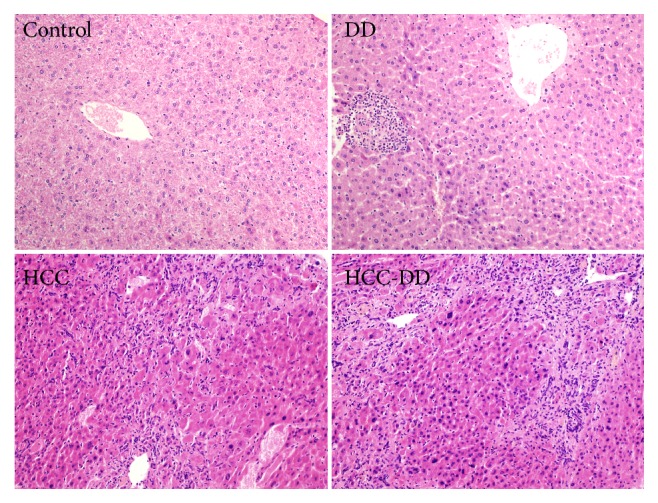
HE stain of the liver tissue. Scale, 200X. Control, Control group; DD, DD group; HCC, HCC group; HCC-DD, HCC-DD group.

The liver tissues in all groups were analyzed at the end of the building of HCC at the 20th week. The hematoxylin-eosin (H&E) staining results showed that the constructions of hepatocytes were in normal situation with full-constructed hepatic lobes, aligned nuclei, radially distributed hepatic cords, and apparent hepatic sinusoids in the Control group and DD group mice. The liver tissue in HCC group and HCC-DD group showed the carcinoma cells were of all sizes, multinucleated tumor giant cells, and funicular slice distributed and accumulated irregularly without the normal hepatocytes constructions. The Edmondson-Steiner classification of the liver tissue in HCC group was the III level while the HCC-DD group was the IV level. The results showed that the malignant degree of the tumor tissue was higher in HCC-DD (Figure 3).

### 3.3. Weight and Survival Analysis

**Figure 4:**
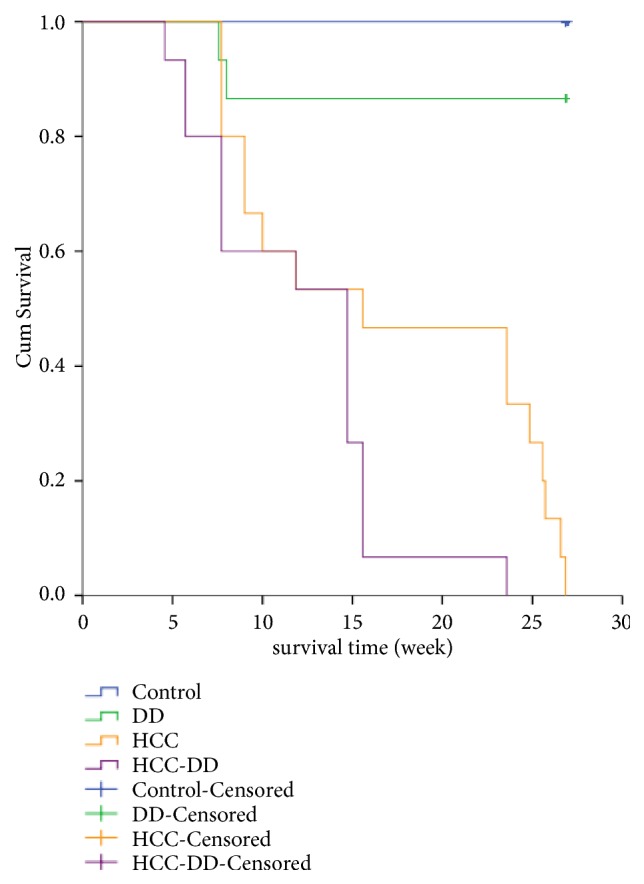
Survival analysis. Kaplan-Meier and Log-Rank (Mantel-Cox) Pairwise Comparison test of survival distributions for different levels of each group. Control-Censored and DD-Censored: when the study was completed on the 20th week, there were alive mice in Control group (*n* = 15) and DD group (*n* = 13), but the survival time was not observed. Control, Control group; DD, DD group; HCC, HCC group; HCC-DD, HCC-DD group.

The spleen weight of the HCC group was the highest, and the stomach weight of the HCC group was the lowest (P<0.05, Table 1). Liver weight and body weight of the HCC group and HCC-DD group were lower than other groups; they were higher in HCC-DD group compared with HCC group because of the increased tumor size inside the HCC-DD liver (P<0.05, Table 1). The survival analysis indicated that the HCC-DD mice have poorer survival rate when compared with the HCC mice (*P*<0.05, Figure 4).

### 3.4. The Expression Levels of Oatp2a1 and Oatp2b1

#### 3.4.1. The mRNA Levels of Oatp2a1 and Oatp2b1

**Figure 5:**
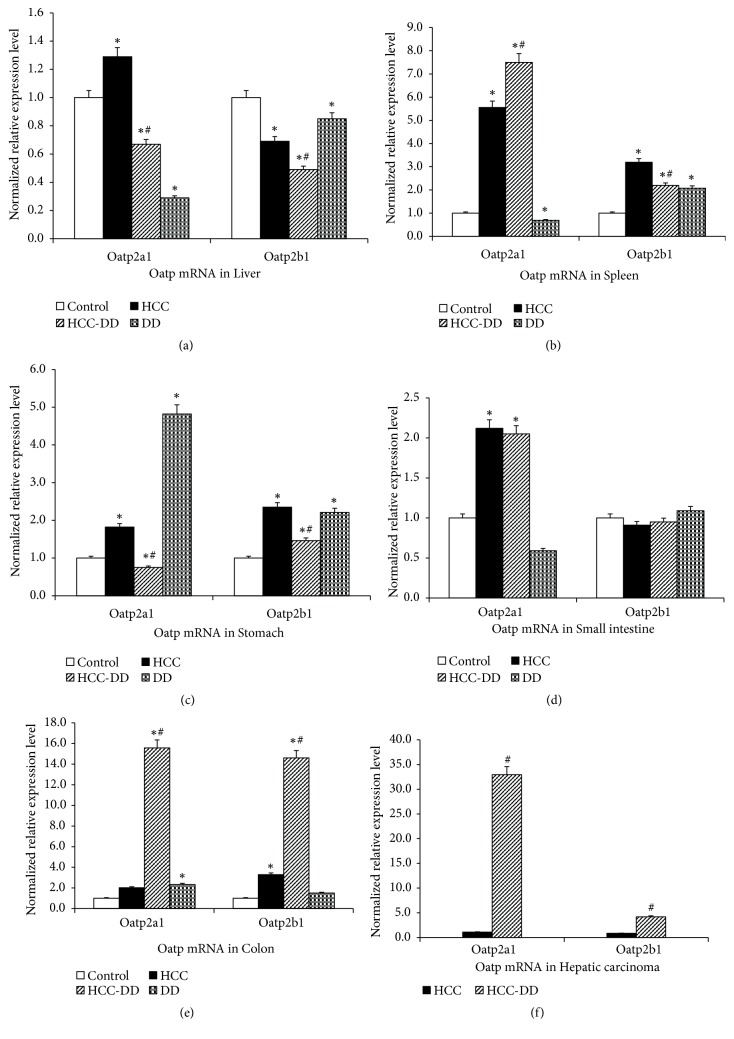
The expression level of Oatp2a1 and Oatp2b1 mRNA in digestive system tissues. (a) liver tissue; (b) spleen tissue; (c) stomach tissue; (d) small intestine tissue; (e) colon tissue; (f) hepatic carcinoma tissue. Control, Control group; DD, DD group; HCC, HCC group; HCC-DD, HCC-DD group. ^*∗*^*P*<0.05 vs. Control group; ^#^*P*<0.05 vs. HCC group.

The expression level of liver Oatp2a1 mRNA in DD group was the lowest in all the groups; it was lower in HCC-DD group compared with HCC group. For the Oatp2b1 mRNA, the expression level was decreased in DD group compared with control group; the expression level was also decreased in HCC-DD group compared with HCC group (P<0.05, Figure 5(a)). In the spleen tissue, the expression level of Oatp2a1 mRNA in HCC-DD group was the highest, while the DD group was the lowest; for Oatp2b1 mRNA, it was the highest in HCC group (P<0.05, Figure 5(b)). In the stomach tissue, the expression levels of Oatp2a1 mRNA and Oatp2b1 mRNA were lower in HCC-DD group compared with HCC group; they were increased in DD group compared with Control group and the expression of Oatp2a1 mRNA in DD group was the highest (P<0.05, Figure 5(c)). In the small intestine tissue, for the Oatp2a1 mRNA, the expression levels in HCC group and HCC-DD group were higher than Control group (P<0.05, Figure 5(d)); however, no difference was found in all groups for Oatp2b1 mRNA. In the colon tissue, for both Oatp2a1 mRNA and Oatp2b1 mRNA, the expression level in HCC-DD group was the highest (P<0.05, Figure 5(e)). Compared with HCC group, the expression of Oatp2a1 mRNA and Oatp2b1 mRNA of hepatic carcinoma was increased in HCC-DD group (P<0.05, Figure 5(f)).

#### 3.4.2. The Protein Levels of Oatp2a1 and Oatp2b1

**Figure 6:**
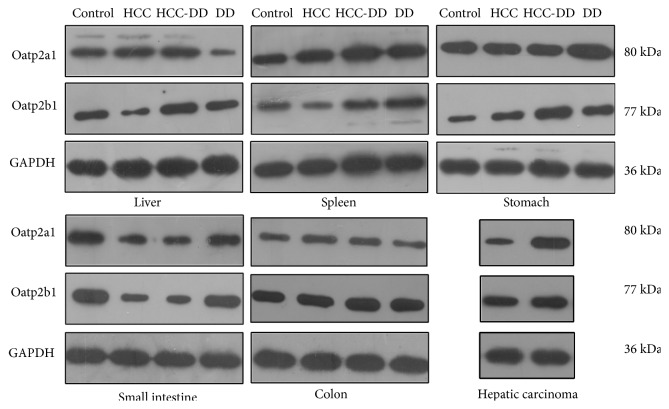
The expression level of Oatp2a1 and Oatp2b1 protein in digestive system tissues. Control, Control group; HCC, HCC group; HCC-DD, HCC-DD group; DD, DD group; GAPDH, glyceraldehyde 3-phosphate dehydrogenase.

**Figure 7:**
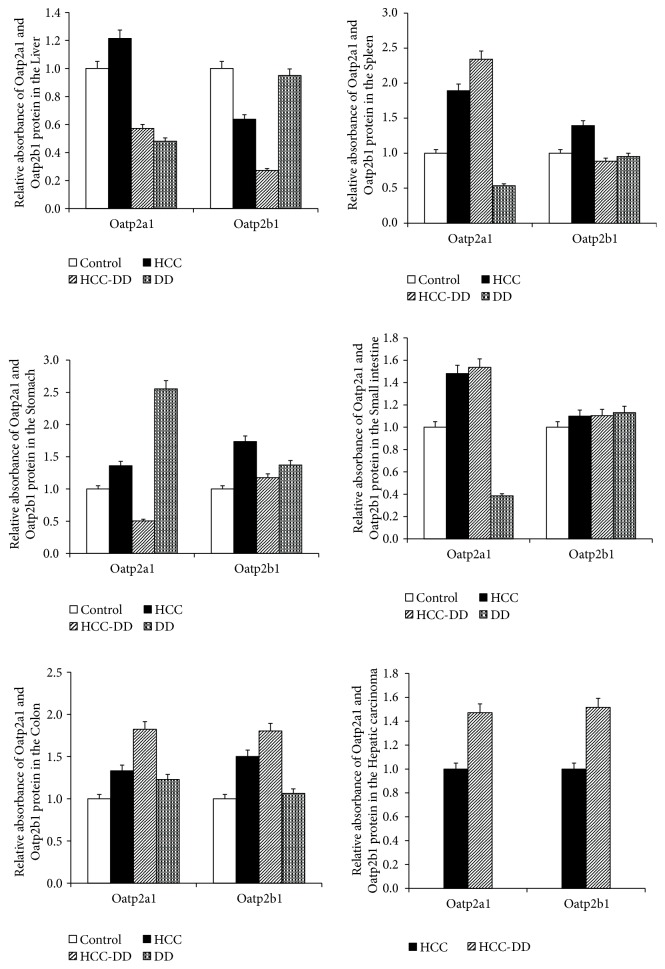
The relative absorbance of Oatp2a1 and Oatp2b1 protein in digestive system tissues. Control, Control group; HCC, HCC group; HCC-DD, HCC-DD group; DD, DD group.

The expression levels of Oatp2a1 and Oatp2b1 in different groups and tissues showed a different trend. The levels of both Oatp2a1 and Oatp2b1 protein were almost consistent with the levels of Oatp2a1 and Oatp2b1 mRNA (Figures 6 and 7). In the liver tissue, the expression levels of Oatp2a1 and Oatp2b1 were decreased in the DD group compared with Control group, and they were decreased in the HCC-DD group compared with HCC group. In the spleen tissue, the expression level of Oatp2a1 protein in DD group was lower than Control group, while it was increased in HCC-DD group compared with HCC group; for the Oatp2b1 protein, the trend was the reversed. In the stomach tissue, the expression level of Oatp2a1 protein and Oatp2b1 protein in DD group was higher than Control group, while they were decreased in HCC-DD group compared with HCC group. In the small intestine tissue, the expression level of Oatp2a1 protein in DD group was lower than Control; however no difference was found in all groups for Oatp2b1 protein. Compared with HCC group, the expression levels of Oatp2a1 and Oatp2b1 in the colon tissue and hepatic carcinoma tissue were increased significant in HCC-DD group.

## 4. Discussion

DD shows relatively high prevalence rates; it is ranked by the World Health Organization (WHO) as the highest global cause of “years of life lived with disability” for all age groups [31]. Cancer patients usually live under chronic stress caused by diagnosis-related strong emotional experience and depression. The study indicated a subsequent risk of DD in patients with HCC, and the risk increased for those with female gender, aged 40 to 59 and aged 60 to 79, with metastasis, with HCV, or with liver cirrhosis [32, 33]. In spite of the fact that no association was detected between DD and risk of HCC in people aged 65 years or older in Taiwan without the data of the younger people, other studies have found an etiological association [12, 34].

Studies have shown that reserpine is widely used to establish mouse depression model [35]. Reserpine is not lethal. Chronic rather than an acute dose of reserpine was used convincingly in the study to induce DD animals. SPT was used for this study, since previous studies have shown that sucrose consumption is a weak and inconsistent index of reward responsiveness in DD [36]. 5-HT1A receptor is closely related to emotional disorders; it is an admittedly important biological markers of DD [37]. This study showed the effect of reduced sucrose consumption in DD group and HCC-DD group at Day 14. Besides, DD group and HCC-DD group had lower body weight and food intake compared with Control group and HCC group. The expression of 5-HT1A receptor in the CA1 region of the hippocampus of the DD mice was decreased compared with the blank mice. These results suggested that DD animal models are successfully established after reserpine administration (0.28 mg/kg i.p.) for 14 consecutive days.

Animal models are crucial tools in the study of HCC. Chemically induced HCC which has an irreversible process characterization by structural DNA changes is closer to human HCC, and DEN induced HCC model is the most frequently used [38]. In this study, DEN and CCl4 were used to establish HCC models as a classic HCC animal model for 20 weeks. The H&E staining results indicated the HCC occurred in the HCC group and HCC-DD group. The survival analysis indicated that there was no significance difference between Control group and DD group in the survival rate, liver weight, spleen weight, stomach weight, and body weight. The results suggested that the effect of DD did not continue for 20 weeks or did not enough affect the mice growth. The survival rate of HCC-DD group was poorer than HCC group. The body weights of the HCC group and HCC-DD group were lower than other groups. And the body weight of HCC-DD group with a higher liver weight was higher than HCC group; it was due to increased tumor size inside the HCC-DD liver. The comparison of survival rate, body weight, and liver weight indicated that DD might be beneficial to the growth of the tumor in HCC-DD mice.

DD and tumor growth could suppress the immune function of mice to different degrees and change the body microenvironment [39]. Immune dysregulation may be a central feature common to DD which induced decreased natural killer cell cytotoxicity and elevated IL-6, TNFalpha, and CRP [40]. At the molecular level, the DD induce the production and secretion of dopamine, catecholamines, and glucocorticoids, which influence the body internal environment and promote proliferation, apoptosis susceptibility, and migration/invasion potential of cancer cells [26]. Recent studies demonstrate that some Oatps are up- or downregulated in several cancers and that Oatp expression might affect cancer development, Oatps could be valuable targets for anticancer therapy [41]. Oatp 2a1 and Oatp 2b1 as members of the solute carrier (SLC) transporters family also undertake the responsibility [20, 21].

Oatp2 is mainly expressed on the sinusoidal membrane of hepatocytes and transporting bilirubin from plasma into the liver. It plays an important role in the excretion of bilirubin and liver toxin. The results showed that, compared with Control group, the expression levels of Oatp2a1 were decreased in liver tissue, spleen tissue, and small intestine tissue in DD group while it increased in colon tissue and stomach tissue; the expression levels of Oatp2b1 were increased in spleen tissue, stomach tissue and colon tissue in DD group. That suggested the DD could regulate the expression of Oatp2a1 and Oatp2b1 in digestive system tissues especial in liver tissue. The expression levels of Oatp2a1 and Oatp2b1 were decreased in liver tissue in HCC-DD group compared with HCC group. However, the study found that downregulation of OATP2 may be involved in the development of hyperbilirubinemia and hepatotoxicity [42]. So, the reason of bigger tumor size and poorer survival rate of HCC-DD mice might be the decreased Oatp2a1 and Oatp2b1 in the internal environment of depression. In other words, the lower level of Oatp2a1 and Oatp2b1 from the DD might be beneficial to the growth of the tumor in HCC-DD mice. And the toxin metabolism disordered by the decreased expression of Oatp2a1 and Oatp2b1 in liver of HCC-DD. The accumulation of toxins in the tissue of hepatocellular carcinoma might result in an increase of expression of Oatp2a1 and Oatp2b1 in compensatory activity, so they were higher in hepatic carcinoma tissue from HCC-DD mice.

## 5. Conclusions

Indeed, there was a positive relationship between DD and the overall occurrence risk of HCC. The pathogenic factors might be an imbalanced body homeostasis caused by DD. At the molecular levels, the decreased expression of Oatp2a1 and Oatp2b1 by DD might be involved. However, further studies are needed to explore the role of the Oatp2a1 and Oatp2b1 played in the progression of cancer.

## Figures and Tables

**Table 1:** Liver weight, spleen weight, stomach weight, and body weight (n=15, mean ± standard deviation).

Group	Liver	Spleen	Stomach	Body weight
Control	1.45 ± 0.06	0.09 ± 0.00	0.21 ± 0.02	26.67 ± 0.84
DD	1.53 ± 0.05	0.11 ± 0.00	0.20 ± 0.01	28.82 ± 1.07
HCC	0.83 ± 0.08*∗*^#^	0.15 ± 0.03*∗*	0.15 ± 0.01*∗*^#^	15.94 ± 0.87*∗*^#^
HCC-DD	1.19 ± 0.07*∗*^#&^	0.11 ± 0.01	0.17 ± 0.01	20.99 ± 1.00*∗*^#&^

Control, Control group; DD, DD group; HCC, HCC group; HCC-DD, HCC-DD group. ^*∗*^*P*<0.05 vs. Control group; ^#^*P*<0.05 vs. DD group; ^&^*P*<0.05 vs. HCC group.

## Data Availability

The datasets used and/or analysed during the current study are available from the corresponding author on reasonable request.
